# Percutaneous Pedicle Screws in the Obese: Should the Skin Incision Be More Lateral?

**DOI:** 10.7759/cureus.4966

**Published:** 2019-06-21

**Authors:** Kyle W Mombell, Jacob E Waldron, Patrick B Morrissey, Nelson S Saldua

**Affiliations:** 1 Orthopaedic Surgery, Naval Medical Center San Diego, San Diego, USA; 2 Orthopaedic Spine Surgery, The Vancouver Clinic, Vancouver, USA

**Keywords:** percutaneous, lumbar fusion, pedicle, lumbar spine, lumbar, obese

## Abstract

Objective: To determine if the skin incision for lumbar percutaneous pedicle screws should be more lateral in the obese patient.

Methods: This was a retrospective radiographic analysis of 30 obese and non-obese lumbar spine computed tomography (CT) radiographs comparing the depth of soft tissue along the anatomic axis of the pedicle at L4 and L5.

Results: The average distance from the pedicle trajectory on the skin to the lateral border of the pedicle at L4 was 1.4 cm and 3.8 cm in the non-obese and obese groups, respectively. The average distance from the pedicle trajectory on the skin to the lateral border of the pedicle at L5 was 2.1 cm and 4.3 cm in the non-obese and obese groups, respectively; both these differences reached statistical significance, p <0.05.

Conclusions: This radiographic study supports a more lateral start point for percutaneous pedicle screws in obese patients to maintain an anatomic trajectory when inserting percutaneous pedicle screws into the lumbar spine at L4 and L5. If a skin incision is made at only 1 cm lateral to the pedicle in the obese patient, the surgeon often has to place significant traction on the skin edge to lateralize their instrumentation to achieve an appropriate angle of insertion. By making a more lateral skin incision, less manipulation of the skin and soft tissues is needed to maintain an anatomic trajectory of the pedicle screw. Decreasing soft tissue manipulation may decrease wound and instrumentation complications in this at-risk population.

## Introduction

Instrumented fusion with lumbar pedicle screw placement has a proven history of forming a rigid construct that results in fusion for a variety of spinal pathologies [1-3]. This can be performed via a standard open surgical approach, or through percutaneous techniques. Recently, minimally invasive procedures have experienced a dramatic increase in popularity in spine surgery because of the significant morbidity involved in a standard open exposure [4, 5].

Percutaneous pedicle screws have demonstrated lower complication rates and significantly less morbidity when compared to the standard open approach to the posterior lumbar spine [2]. However, the technique is not without its challenges. One fundamental limitation noted among many spine surgeons is the learning curve present with switching from an open to percutaneous approach [6]. A significant problem contributing to the learning curve of percutaneous instrumentation is that visualization is severely limited and almost exclusively guided by intraoperative fluoroscopy. Several authors [3, 7] have described techniques to familiarize the surgeon with an accurate cutaneous starting point; inter-patient variability such as anatomy, level(s) of disease, pathology treated, deformity present, trauma, and soft tissue variability can make the procedure substantially more difficult.

Increasing rates of obesity are well described [8,9]. It is essential to consider the anatomic variation the obese patient presents when planning for percutaneous instrumentation in the lumbar spine. The procedure can be more technically challenging in obese patients due to greater difficulty with obtaining adequate fluoroscopic imaging, as well as the greater depth of dissection needed [7]. Obesity is associated with significant increases in postoperative complications following lumbar spine surgery; however, there is no known increased risk of complications in obese patients undergoing minimally invasive spine surgery (MISS) procedures [8-10]. MISS may, therefore, be a reasonable consideration for many surgeons during the preoperative discussion with obese patients to avoid the substantially increased amount of soft tissue dissection required in an open procedure.

Harris et al. [3] described an operative technique for percutaneous pedicle screws in the lumbar spine with a skin start point 1 cm from the midline of the pedicle on a posterior-anterior (PA) intraoperative fluoroscopy image. This allows for the medializing trajectory of the pedicle screws required to maintain a collinear relationship with the pedicle. While a more lateral skin incision is mentioned in the literature [7], no studies quantify the difference. We sought to identify the difference in cutaneous incision site/ pedicle screw start point in obese (body mass index (BMI) >30 kg/m2) patients compared with patients of a normal BMI (18.5-25kg/m2). In obese patients, we hypothesized that because of the greater depth of soft tissue, the start point should be more lateral to achieve an anatomic screw trajectory within the pedicle.

## Materials and methods

The study protocol was approved by the Naval Medical Center San Diego Institutional Review Board in compliance with all applicable federal regulations governing the protection of human subjects. The patient radiology viewing software, Carestream PACS (Carestream Health, Inc., NY USA), at our institution was searched for sequential computed tomography (CT) scans of the abdomen/pelvis. CT scans of the abdomen/pelvis were chosen over magnetic resonance imaging (MRI) to ensure the cutaneous margin was included in the study. The electronic health record was cross-referenced for the patient’s BMI. Thirty CT scans were identified from patients with a BMI greater than 30 kg/m2 (obese) and 30 scans were identified from patients with a BMI 18.5-25 kg/m2 (normal).

**Figure 1:**
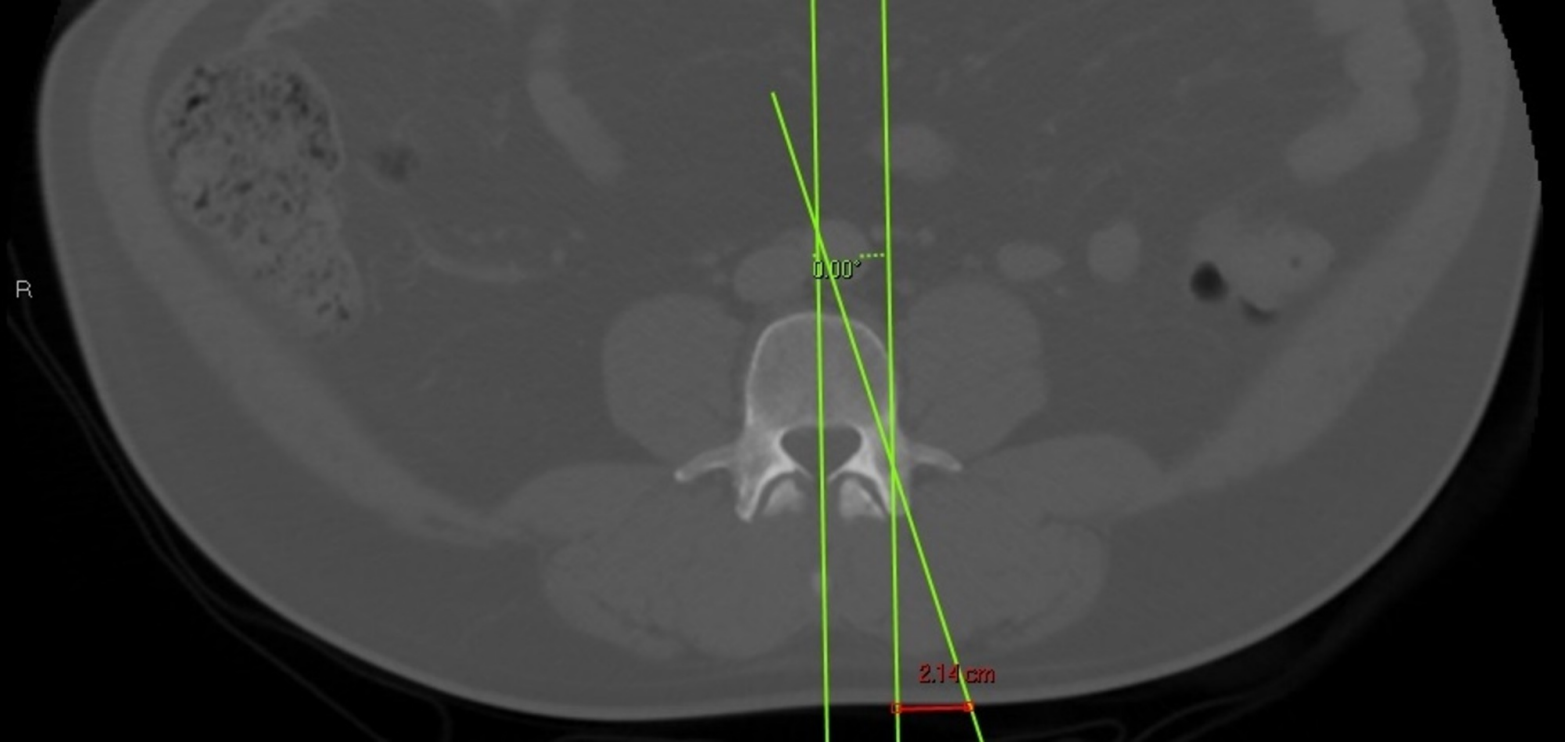
Red line depicting the measurement between the lateral border of the pedicle and the pedicle trajectory

Axial CT scan slices were identified at both L4 and L5 levels in each patient using the PACS software. Care was taken to cross-reference each axial image with sagittal images to confirm the appropriate level. A slice was then identified at both L4 and L5 on the axial view which was centered in the pedicle and collinear with the pedicle’s sagittal trajectory. A line was drawn approximating the anatomic path of the pedicle on the axial images using the digital measurement tools. This line was extended to the skin. A digital plumb line was also placed at the lateral aspect of the pedicle to represent the lateral point of the pedicle on a PA fluoroscopic image. This line was made in parallel with a line bisecting the vertebral body to minimize the effect of patient positioning (Figure 1). The distance on the skin from the lateral aspect of the pedicle to the anatomic trajectory of the pedicle was measured. Statistical analysis using a t-test was performed to determine any differences in skin start points to maintain trajectories collinear with the pedicles for both L4 and L5 in the obese and non-obese BMI groups.

## Results

Thirty subjects were identified in the obese and normal BMI groups. The normal BMI group had an average BMI of 22.6 kg/m2 and the obese group had an average BMI of 35.15 kg/m2. The patient demographics were similar between the obese and non-obese groups as depicted in Table 1. The average distance from the pedicle trajectory on the skin to the lateral border of the pedicle at L4 in the non-obese group was 1.4 cm compared to 3.8 cm in the obese group; this was statistically significant, p <0.01. The average distance at L5 was 2.1 cm and 4.3 cm in the non-obese and obese groups, respectively; this was also found to be statistically significant, p <0.01.

**Table 1:** Summary of patient demographics including BMI, age, and sex

		Obese (n=30)	Normal (n=30)
BMI (kg/m^2^)	Average	35.15	22.6
	Range	30.2 - 46.07	19.46- 24.94
Age (years)	Average	42.9	48.07
	Range	26 - 68	20 - 91
Sex	Male	13	12
	Female	17	18

## Discussion

Percutaneous pedicle screws are widely used and have been shown to decrease patient morbidity and lower complication rates when compared to open techniques while still reliably forming a rigid construct [2]. However, this technique presents its own unique complexities including a significant learning curve and difficulty with imaging. Performing minimally invasive surgical techniques on the obese patient can be particularly challenging due to the depth of dissection necessary and the larger soft tissue envelope obscuring fluoroscopic imaging. Despite this, no differences in surgical technique are widely utilized for the obese population.

This radiographic study sought to compare the most ideal skin incision to maintain an anatomic pedicle screw trajectory in the obese versus non-obese patient. The findings support a more lateral skin start point for percutaneous pedicle screws in obese patients to maintain an anatomic trajectory when instrumenting the L4 and L5 levels.

Previous technique and review articles [3, 7] have commented on the recommended skin incision for percutaneous pedicle screws as 1 cm lateral to the pedicle. No known comparison or studies exist in the literature for skin incision location between obese and non-obese patients. This study represents the first of its kind to evaluate this potential effect of obesity on the skin start points for percutaneous pedicle screw instrumentation. If the skin incision is made only 1 cm lateral to the pedicle in the obese patient, the surgeon often has to place significant traction on the skin edges to lateralize their instrumentation and achieve an appropriate angle of screw insertion. If the surgeon is unable to appropriately medialize the screw trajectory, he or she risks breaching the lateral cortex. By making a more lateral skin incision, less manipulation of the soft tissues is needed to medialize the screw trajectory and achieve an anatomic screw alignment. A more lateral skin incision may also decrease wound complications in the obese population by reducing the traction on the incision. Obese patients are traditionally at a higher risk of surgical site infections and careful soft tissue handling is an important adjunct to preventing these complications.

Further research could identify a clinical difference in reducing infection rates or instrumentation complications such as lateral breaches when a more lateral skin incision is used in the obese patient undergoing percutaneous pedicle screw placement.

## Conclusions

This study supports a more lateral skin incision for percutaneous pedicle screws in the lumbar spine of obese patients. Further clinical research is necessary to validate the clinical and technical benefits of minimizing traction to the skin in this higher-risk patient population.
